# Clinical Significance and Microbiological Characteristics of *Staphylococcus lugdunensis* in Cutaneous Infections

**DOI:** 10.3390/jcm13154327

**Published:** 2024-07-24

**Authors:** Dimitra Koumaki, Sofia Maraki, Georgios Evangelou, Evangelia Rovithi, Danae Petrou, Erato Solia Apokidou, Stamatios Gregoriou, Vasiliki Koumaki, Petros Ioannou, Kyriaki Zografaki, Aikaterini Doxastaki, Kalliopi Papadopoulou, Dimitra Stafylaki, Viktoria Eirini Mavromanolaki, Konstantinos Krasagakis

**Affiliations:** 1 Dermatology Department, University Hospital of Heraklion, 71110 Heraklion, Greece; gevag600@yahoo.co.uk (G.E.); eva.rovithi@gmail.com (E.R.); danaepetrou@yahoo.gr (D.P.); kikizog@yahoo.gr (K.Z.); katerina.doxastaki@gmail.com (A.D.); krasagak@uoc.gr (K.K.); 2 Department of Clinical Microbiology, University Hospital of Heraklion, 71110 Heraklion, Greece; sofiamaraki@yahoo.gr (S.M.); stafylaki.dimitra@gmail.com (D.S.); 3 Department of Internal Medicine, Agios Nikolaos General Hospital, Knosou 4, 72100 Agios Nikolaos, Greece; erato_97@hotmail.com; 4 1st Department of Dermatology and Venereology, Medical School of Athens, National and Kapodistrian University of Athens, Andreas Sygros Hospital, I. Dragoumi 5, 16121 Athens, Greece; stamgreg@yahoo.gr; 5 Department of Medical Microbiology, Medical School of Athens, National and Kapodistrian University of Athens, 75 Mikras Asias Str., Goudi, 11527 Athens, Greece; vkoumaki@gmail.com; 6 Department of Internal Medicine, University Hospital of Heraklion, 71110 Heraklion, Greece; 7 School of Medicine, University of Crete, 70003 Heraklion, Greece; 8 2nd Department of Internal Medicine, General Hospital of Venizeleio, Knossou Avenue 44, 71409 Heraklion, Greece; kalliopi1997@hotmail.gr; 9 Department of Paediatrics, Agios Nikolaos General Hospital, 72100 Agios Nikolaos, Greece; virnaball@gmail.com

**Keywords:** *Staphylococcus lugdunensis*, antibiotic resistance, penicillin, erythromycin, skin infections

## Abstract

**Background/Objectives:** *Staphylococcus lugdunensis* is a coagulase-negative staphylococcus (CoNS) commonly found on human skin. Unlike other CoNS, *S. lugdunensis* has a notable potential to cause severe infections comparable to *Staphylococcus aureus*. This study aimed to characterize the clinical and microbiological profile of patients with *S. lugdunensis* skin infections at a single center. **Methods:** We conducted a retrospective analysis of patient records from the Dermatology Department of the University Hospital of Heraklion, Greece, covering the period from January 2014 to January 2024. Patients’ clinical presentations, demographics, infection sites, comorbidities, prior infections, antimicrobial treatments, and therapeutic responses were examined. Specimens were collected, transported, and processed according to standardized microbiological protocols. Bacterial identification and antibiotic susceptibility testing were performed using the Vitek 2 automated system and MALDI-TOF MS, with results interpreted according to Clinical and Laboratory Standards Institute (CLSI) criteria. **Results:** A total of 123 skin specimens positive for *S. lugdunensis* were analyzed. The cohort comprised 62 males (50.4%) and 61 females (49.6%), with a mean age of 40.24 ± 20.14 years. Most specimens were collected from pus (84%), primarily from below the waist (66.7%). Hidradenitis suppurativa (26%) was the most common condition associated with *S. lugdunensis*, followed by folliculitis, abscesses, ulcers, cellulitis, and acne. Co-infections with other bacteria were noted in 49.6% of cases, and 25.2% of infections were nosocomially acquired. The majority of patients (65%) received systemic antibiotics, predominantly amoxicillin/clavulanic acid, cefuroxime axetil, and doxycycline, with a cure rate of 100%. All isolates were susceptible to several antibiotics, though resistance to penicillin (28.5%) and clindamycin (36%) was observed. **Conclusions:** *S. lugdunensis* is a significant pathogen in skin infections, capable of causing severe disease. The high cure rate demonstrates the effectiveness of appropriate antibiotic therapy. Continued monitoring and antimicrobial stewardship are essential to manage resistance and ensure effective treatment.

## 1. Introduction

Following its initial description in 1988, *Staphylococcus lugdunensis* was identified from other coagulase-negative staphylococcal species by DNA-relatedness analyses utilizing 11 clinical strains [1]. The Latin name for Lyon is Lugdunum, and the new species bears this name in honor of the French city where the organism was originally isolated [2]. Because of its tendency to cause aggressive native valve infective endocarditis and its resistance to a wide range of antimicrobial treatments, *S. lugdunensis* stands apart among coagulase-negative staphylococci (CoNS). Thirty-five years ago, Freney et al. published the first description of *S. lugdunensis*, a CoNS [3,4]. It results in serious infections comparable to those caused by *Staphylococcus aureus*, particularly acute endocarditis in both native and prosthetic valves [3]. There have also been reports of infections such as osteomyelitis, peritonitis, intravascular catheter infections, infections in prosthetic joints, and urinary tract infections [2,3,4,5,6]. It has been shown that *S. lugdunensis* is also a significant contributor to skin and soft tissue infections (SSTIs), mostly in the groin and mammae [2]. While it can exist harmlessly on human skin, similar to other CoNS, it also has the potential to become a dangerous pathogen, such as in cases of infective endocarditis [4,5,6]. Uniquely, *S. lugdunensis* is capable of causing severe infections similar to those often associated with the more virulent *S. aureus* [2,7,8,9]. Moreover, most isolates of *S. lugdunensis* are still susceptible to a wide range of antimicrobial agents [2,7,8,9,10,11]. Since some clinical laboratories do not regularly specify CoNS, the frequency of *S. lugdunensis* infection is likely underestimated [12,13]. However, the use of matrix-assisted desorption ionization–time of flight mass spectrometry (MALDI-TOF MS) in clinical laboratories may increase the detection and reporting of this species [14,15]. Speciation should be triggered by virulent infection caused by CoNS; in contrast to *S. epidermidis*, *S. lugdunensis* should normally be considered a real pathogen. Of the clinical infections caused by *S. lugdunensis*, 25 to 80 percent are soft tissue infections [16,17,18,19]. According to a number of studies, *S. lugdunensis* typically colonizes and infects areas below the waist [12,20,21,22,23,24]. There are instances of *S. lugdunensis* infective endocarditis (IE) in the context of vasectomy, perineal lesions, and prostate cancer, all of which point to the bacteria’s propensity to colonize and infect the perineal region [25,26,27,28,29,30]. Seventy-three percent of infections (mainly soft tissue abscesses) among 38 patients infected with *S. lugdunensis* in one series happened below the waist [24]. About 20 percent of a group of 140 patients had *S. lugdunensis* colonization in their inguinal region [29]. It seems that inguinal colonization predisposes one to infection following invasive procedures, and shaving the inguinal region before surgery has been linked to a higher incidence of *S. lugdunensis* infections [30,31,32,33,34,35]. Another significant location for *S. lugdunensis* infection is the breast. Non-lactating women have been observed to suffer breast abscesses (both spontaneous and surgical infections) [30]. 

In this study, we aimed to characterize the clinical and microbiological profile and treatment outcomes of patients from whom *S. lugdunensis* was isolated from skin infections at a single center.

## 2. Materials and Methods

### 2.1. Paptients’ Characteristics

Inclusion of Consecutive Patients:

We included all patients who were diagnosed with SSTIs caused by *S. lugdunensis* over a specified period (from January 2014 to January 2024). The diagnoses were confirmed by culture and identification of the bacterium in the laboratory.

Exclusion of Pre-treatment Resistance Factor:

The study did not specifically select patients based on resistance to previous therapy. Instead, the inclusion was purely based on the presence of *S. lugdunensis* in their samples. We aimed to include a broad range of cases to avoid selection bias and to ensure that our findings were representative of the general population affected by *S. lugdunensis*.

Clinical Setting and Patient Characteristics:

Patients were sourced from multiple clinics within our hospital system, including dermatology, general surgery, and emergency medicine departments. This multi-clinic approach was intended to encompass a diverse patient population, each with varying backgrounds and health conditions, rather than limiting the study to a specific subset of patients.

Sample Testing and Collection:

All samples tested were taken as part of routine clinical care, not based on resistance to previous treatments. Tampon testing or similar interventions were not a criterion for inclusion. Each sample was subjected to the same laboratory procedures to isolate and identify *S. lugdunensis*.

The Rationale for This Approach:

The primary objective of our study was to understand the clinical characteristics, outcomes, and microbial profiles of *S. lugdunensis* SSTIs across a broad patient population. By including all consecutive cases without preselecting based on treatment resistance, we aimed to capture the full spectrum of infections and provide a comprehensive overview of the pathogen’s impact.

Patient Classification: Patients were classified into two groups based on their treatment status:

Treated Group: 

This group included patients who received either topical or oral antibiotics and exhibited a significant clinical improvement, defined as the complete resolution of symptoms such as erythema, edema, and pain within a specified follow-up period of three months.

Not Treated Group: 

This group comprised patients who either did not receive any antibiotic treatments or failed to respond to a treatment, as evidenced by the persistence or exacerbation of symptoms.

### 2.2. Sample Collection, Transport, and Processing

After cleansing the skin using 2% chlorhexidine gluconate solution combined with 70% isopropyl alcohol to prevent contamination by superficial bacteria, pus and exudates were collected using a sterile cotton swab, which was immediately placed in Amies transport medium (bioMérieux SA, Marcy L’ Etoile, France). The samples were quickly transported to the Microbiology Laboratory through a pneumatic tube system and were processed within 30 min. Each specimen underwent wet mount preparation, Gram staining, and culturing according to laboratory protocols.

For isolating bacterial pathogens, specimens were inoculated onto Columbia agar with 5% sheep blood, chocolate agar with polyvitex, mannitol salt, Drigalski lactose, and Sabouraud agar plates and incubated aerobically at 36 °C. The samples were also cultured on Shaedler agar with 5% sheep blood at 36 °C under anaerobic conditions. The enriched media provided the necessary nutrients for a broad range of bacteria, while the selective and differential media allowed for the isolation and identification of specific bacterial species by inhibiting the growth of non-target organisms and differentiating between similar species based on their metabolic characteristics.

Bacterial species identification was conducted using standard biochemical tests and the Vitek 2 automated system, which offers rapid and accurate identification by analyzing the metabolic and enzymatic activities of the bacteria [31]. This initial identification was further confirmed with MALDI-TOF MS version 3.2 (both from bioMérieux SA), a sophisticated technique that measures the unique protein profiles of the bacteria, providing highly specific identification.

The Vitek 2 system also performed antibiotic susceptibility testing, determining the sensitivity of the bacterial isolates to various antibiotics. The results were interpreted according to the 2023 Clinical and Laboratory Standards Institute (CLSI) criteria, ensuring that the antibiotic recommendations were based on the latest standards and guidelines [36,37,38].

To ensure accuracy and reliability of the results, quality control strains were used throughout the testing process. These included *Escherichia coli* ATCC 25922, *Pseudomonas aeruginosa* ATCC 27853, *S. aureus* ATCC 25923, and *Enterococcus faecalis* ATCC 29212. These control strains are well-characterized and provide a benchmark for comparing the performance of the identification and susceptibility testing methods.

### 2.3. Statistics

Descriptive analysis and statistical correlations were conducted using IBM SPSS Statistics for Windows (Version 25.0. Armonk, NY, USA: IBM Corp.). The data were meticulously checked for consistency and completeness before analysis. Participants with missing data for a specific variable were excluded from the analysis for that variable. Frequency distributions were generated for categorical variables to understand the prevalence and distribution of different characteristics within the study population. For continuous variables, we conducted exploratory data analysis to identify any potential outliers or anomalies that could impact the results. The results of these analyses were interpreted with caution, considering the sample size and the potential impact of missing data. To ensure transparency and reproducibility, all steps of the data analysis process were documented thoroughly. 

## 3. Results

**Figure 1:**
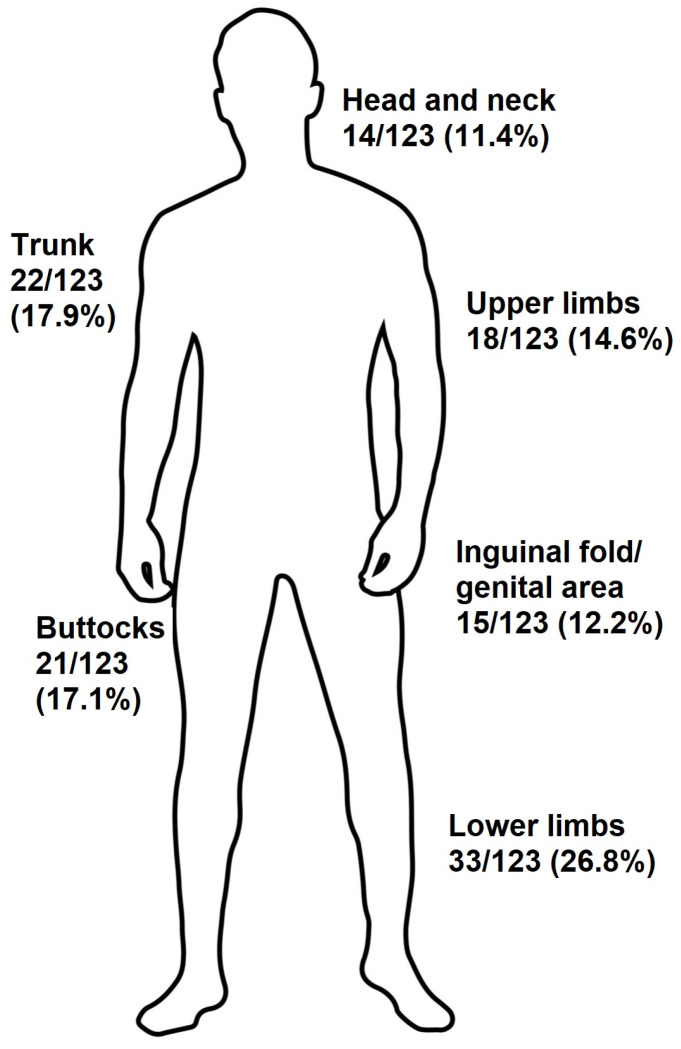
Topography of affected areas affected by *Staphylococcus lugdunensis*.

Between January 2014 and January 2024, 123 skin specimens yielding positive results for *S. lugdunensis* were collected at the Dermatology Department of the University Hospital of Heraklion, Heraklion, Greece. Among the patients, 62 out of 123 (50.4%) were male, and 61 out of 123 (49.6%) were female. The mean age was 40.24 ± 20.14 years. Of the specimens, 104 out of 123 (84%) were collected from pus, 16 out of 123 (13%) from exudates, and 3 out of 123 (2.4%) from tissue cultures. One-third of the patients, 30.9% (38/123), were immunosuppressed, while the other two-thirds of the patients, 69.1% (85/123), were immunocompetent. Clinical findings are presented in Table 1. The majority of specimens with *S. lugdunensis*, 82 out of 123 (66.7%), were collected from below the waist. The highest frequency of *S. lugdunensis* bacteria was observed in the lower limbs (33/123, 26.8%), followed by the trunk (22/123, 17.9%) and buttocks (21/123, 17.1%) (Figure 1). Most patients from whom *S. lugdunensis* was isolated had hidradenitis suppurativa (HS): 32 out of 123 (26%), followed by folliculitis: 17 out of 123 (13.8%), abscesses: 18 out of 123 (14.6%), ulcers: 13 out of 123 (10.6%), cellulitis: 10 out of 123 (8.1%), and acne: 8 out of 123 (6.5%) (Table 1). In 61 out of 123 (49.6%) cases, other bacteria were also isolated. Other bacteria found in the 123 samples where *Staphylococcus lugdunensis* was isolated included *S. aureus, S. caprae*, *S. cohnii, S. epidermidis, S. haemolyticus, S. hominis, Acinetobacter lwofii, Cutibacterium acnes, Enterobacter aerogenes, E. cloacae, and E. faecalis*, as described in detail in Table 2.

*S. lugdunensis* was hospital-acquired in only 31 out of 123 (25.2%) cases. All bacterial isolates were susceptible to linezolid, moxifloxacin, rifampicin, teicoplanin, tigecycline, trimethoprim/sulfamethoxazole, and vancomycin (Table 3). The highest rates of resistance were observed for clindamycin in 36/123 (36%) isolates, followed by penicillin in 35/123 (28.5%), erythromycin in 32/123 (26%), tobramycin in 9/123 (7.3%), tetracycline in 8/123 (6.5%), oxacillin in 6/123 (4.9%), gentamicin and fosfomycin in 5/123 (4.1%), and levofloxacin in 2/123 (1.6%). The majority of patients, 80 out of 123 (65%), received systemic antibiotic treatment, while 43/123 (35%) received only topical treatment. Among those receiving topical treatment, 21/123 (17.1%) used topical fusidic acid, 10/123 (8.1%) used topical mupirocin, and 12/123 (9.8%) used topical clindamycin. Most of these patients received oral amoxicillin/clavulanic acid: 28 out of 123 (22.8%), followed by cefuroxime axetil: 19 out of 123 (15.4%), and oral doxycycline: 18 out of 123 (14.6%). The cure rate was 123 out of 123 (100%), independent of the antibiotic susceptibility of the bacterial isolates, the presence of other bacteria, immunological status, or age of the patients. 

There was no statistically significant correlation between clinical presentation, demographic data, immunological status of patients, topography of skin lesions, antibiotic susceptibility, and treatment outcome.

## 4. Discussion

The present study provides a comprehensive analysis of *S. lugdunensis* skin infections at the Dermatology Department of the University Hospital of Heraklion in Greece over ten years, presenting one of the largest case series on *S. lugdunensis* and skin and soft tissue infections. In our study, all bacterial isolates were susceptible to linezolid, moxifloxacin, rifampicin, teicoplanin, tigecycline, trimethoprim/sulfamethoxazole, and vancomycin. The highest resistance rates were observed with clindamycin in 36/123 isolates (36%), followed by penicillin in 35/123 (28.5%), erythromycin in 32/123 (26%), tobramycin in 9/123 (7.3%), tetracycline in 8/123 (6.5%), oxacillin in 6/123 (4.9%), and gentamicin and fosfomycin each in 5/123 (4.1%). Levofloxacin showed the lowest resistance rate with 2/123 isolates (1.6%). This is consistent with previous studies that show a good antibiotic susceptibility of *S. lugdunensis* [2,8,9].

In our study, penicillin resistance in *S. lugdunensis* was reported at 28.5%, which is higher than that reported in studies from northern Europe, 25.4% in Sweden [39], and 20% in Denmark [40] but lower than studies from Northern Greece 51% [41], the USA 45% [19], and Taiwan 87% [42] and 68.8% [43]. Two studies conducted in Örebro, Sweden, reported penicillin G resistance at 15.5% in 2006 [17], while another team from the same city in 2019 reported a resistance rate of 25.4% [39]. In Denmark in 2009, penicillin resistance in *S. lugdunensis* was reported in 20% of isolates [42]. In the USA, penicillin resistance was reported at 45% [15], and in Taiwan at 87% in 2014 [42] but 68.8% in 2015 [43]. 

Our study reported an erythromycin resistance rate of 26%, which is similar to the rate reported in Denmark in 2009 (26%) but higher than rates reported in Northern Greece [41], Sweden (6.3%) [39], Virginia, USA (9%) [19], and Taiwan (17% in 2014 and 25% in 2015) [42,43]. Our study shows the highest rates of clindamycin resistance in *S. lugdunensis* at 36%, which is higher than the rates reported in Sweden (8.5%), Virginia, USA (3%) [19], and two studies from Taiwan, which reported resistance rates of 17% [42] and 18% [43]. 

The 4.9% resistance to oxacillin is consistent with previous reports indicating that methicillin resistance in *S. lugdunensis* remains relatively low. This is a positive finding, as it suggests that most strains remain susceptible to methicillin and other beta-lactams [39].

The observed resistance rate of 36% to clindamycin is significant, as clindamycin is often used for treating skin and soft tissue infections caused by staphylococci. This rate is higher than what has been reported in some studies, where clindamycin resistance in *S. lugdunensis* has been noted to be less than 10% in certain populations [17,39]. The emergence of higher resistance rates could indicate a shift in susceptibility patterns that warrants attention.

Unlike other CoNSs, *S. lugdunensis* is still highly responsive to the majority of antibiotics, especially beta-lactams. The only antibiotic that produces wildly inconsistent results and a resistance level of roughly 50%, depending on the study, is Fosfomycin [10,11]. The fusB gene is primarily responsible for this resistance. According to the study, beta-lactamase production varies as well, being reported in between 0% and 70% of the strains. Methicillin resistance still seems to be marginal in *S. lugdunensis*, despite the fact that only some strains have the mecA gene linked to methicillin resistance. This phenotype is common among staphylococci, though. Kleiner et al. discovered that 3% of the 36 strains examined in the biggest collection of bacteria examined for methicillin resistance and the mecA gene also exhibited oxacillin resistance [19]. The European Committee on Antimicrobial Susceptibility Testing (EUCAST) and the CLSI in the United States both have guidelines that state that *S. aureus* and *S. lugdunensis* have clinical breakpoints that are higher than those of other CoNS [37,38]. The mecA gene makes these two species, which have oxacillin MIC values of >2 mg/L, largely resistant to methicillin. Since a lower breakpoint accurately classifies most CoNS with the mecA gene while overcalling resistance for *S. lugdunensis*, the equivalent MIC for other CoNS is >0.25 mg/L [38]. Cefoxitin has emerged as a more trustworthy marker for methicillin resistance, which is noteworthy. Additionally, *S. aureus* and *S. lugdunensis* have comparable breakpoints. Methicillin resistance and the existence of the mecA gene are predicted by an MIC threshold of cefoxitin ≥ 8 mg/L [37]. Therefore, *S. lugdunensis* does not appear to share resistance genes through horizontal genetic transfer, despite its presence in nosocomial infections and its colocalization on the skin with other CoNSs and *S. aureus*, which is frequently methicillin-resistant. However, a number of mobile genetic components have been highlighted in recent genomic investigations. Tse et al. completed the entire sequencing of *S. lugdunensis* [16].

The demographic distribution showed an almost equal prevalence among male and female patients, with a slight male predominance (50.4% males vs. 49.6% females). The mean age of 40.24 years aligns with previous studies indicating that *S. lugdunensis* infections are not restricted to a specific age group. The majority of isolates were obtained from pus (84%), reflecting the organism’s propensity to cause suppurative infections. Previous studies have indicated that *S. lugdunensis* infections commonly occur below the waist, particularly in the groin region [17,19]. Our study corroborates these findings as the majority of *S. lugdunensis* isolates were obtained from sites below the waist, which may correlate with the higher incidence of certain conditions such as hidradenitis suppurativa (HS) in these areas [21]. Regions containing apocrine sweat glands are often the most commonly infected sites. It has been proposed that, like *Staphylococcus aureus* and *Staphylococcus sciuri, S. lugdunensis* may reside as a commensal organism in these areas, which include the axilla, anogenital region, auditory canals, eyelids, and mammary areolae. Areas under the waist, such as the groin and perineal regions, tend to be more moist due to sweat accumulation, providing an environment where *S. lugdunensis* thrives [39]. The higher density of sebaceous glands under the waist produces sebum, which can provide nutrients that support the growth of certain bacteria, including S. lugdunensis. Additionally, clothing and physical activity can cause occlusion and friction in these areas, leading to minor skin abrasions and an environment conducive to bacterial colonization [21]. HS was the most common clinical condition associated with *S. lugdunensis* (26%), followed by folliculitis, abscesses, ulcers, cellulitis, and acne. 

HS is a chronic inflammatory skin condition characterized by painful nodules, abscesses, and sinus tracts primarily affecting apocrine gland-rich areas such as the axillae, groin, and perianal regions [34,41,44]. The exact etiology of HS remains unclear, but the disease involves significant dysregulation of both innate and adaptive immune responses. *S. lugdunensis* is often found in higher abundance in HS lesions compared to other skin conditions, likely due to the unique microenvironment of HS lesions, which may favor the colonization and persistence of *S. lugdunensis* [34,45,46,47]. The chronic inflammation and tissue damage in HS can lead to increased availability of nutrients and niches that support the growth of S. lugdunensis. Additionally, the recurring nature of HS lesions may disrupt normal skin barriers, facilitating bacterial invasion and persistence. *S. lugdunensis* possesses several virulence factors that help it evade the immune system and establish infection. It produces a variety of exoenzymes and toxins, including lipases, proteases, and hemolysins, which can damage host tissues and immune cells. Furthermore, *S. lugdunensis* can form biofilms, which protect the bacteria from immune responses and antibiotic treatment. One specific immune evasion strategy involves the production of the enzyme coagulase, which leads to the formation of a fibrin clot around the bacteria, shielding it from phagocytosis. Although *S. lugdunensis* coagulase activity is generally weaker than that of S. aureus, it still contributes to its ability to persist in the host [40,41,42,43,44]. Studies have shown that the immune response to *S. lugdunensis* involves both the innate and adaptive immune systems. Neutrophils play a critical role in the initial response to *S. lugdunensis* infection, attempting to phagocytose and kill the bacteria. However, the presence of biofilms and protective exoenzymes can hinder this process. Adaptive immune responses are also activated, with T cells and antibodies targeting *S. lugdunensis* [38]. However, the effectiveness of these responses can be variable, and chronic infections like those seen in HS can lead to immune exhaustion and reduced efficacy of the adaptive immune system. 

Biofilm development is a hallmark of HS, and it may play a crucial role in inducing the host pathophysiological response. The prospect of using the microbiome as a biomarker for HS has increased with the discovery of particular microbial signatures linked to the illness [47]. Microbiome-based biomarkers that can help with the diagnosis and stratification of HS patients may be developed by examining the relative abundance and composition of important bacterial taxa. Numerous investigations have demonstrated the existence of inflammatory markers in HS, and it is recognized that the presence or lack of particular bacteria may have an impact on these markers’ expression. HS has been linked to obesity, leading researchers to investigate how body composition might affect the microbiome in this condition. Obese patients with HS often show dysbiosis, marked by microbial diversity, composition, and function imbalances. These microbial changes are believed to potentially play a role in the persistent inflammation seen in HS. In contrast, nonobese HS patients have unique microbiome profiles compared to their obese counterparts. Although there might still be some dysbiosis, the particular microorganisms affected and the level of microbial disturbances may vary [44].

The 100% cure rate observed in our study of 123 patients with *S. lugdunensis* skin infections warrants careful consideration. Several factors contribute to this outcome. The study included patients with confirmed *S. lugdunensis* infections that were accurately diagnosed and treated. The careful selection of patients likely minimized confounding factors that could affect treatment outcomes. All patients received targeted antibiotic therapy based on susceptibility testing, which is crucial for achieving high cure rates. The majority of *S. lugdunensis* strains are susceptible to beta-lactams, significantly contributing to the successful treatment. Most infections in our cohort were classified as mild to moderate, which typically respond well to antibiotic treatment. The nature of these infections likely influenced the high cure rate. Additionally, patients were closely monitored throughout the treatment process, allowing for timely interventions when necessary. This proactive approach likely contributed to the overall success of the treatment.

In a study involving 75 healthy individuals, *S. lugdunensis* was isolated from 50 subjects, indicating a significant presence [48]. The bacterium was predominantly found in the lower abdomen and extremities, particularly in areas such as the groin and the nail bed of the first toe. In contrast, *S. aureus* was less frequently isolated from these sites, suggesting that *S. lugdunensis* occupies distinct niches in the skin flora compared to S. aureus [48,49]. It has also been reported that *S. lugdunensis* can colonize the skin of up to 67% of individuals, particularly in moist areas like the inguinal fold and perineum. Although *S. lugdunensis* is primarily a skin commensal, it can also lead to infections, particularly skin and soft tissue infections [50]. This dual role underscores its significance in both health and disease contexts. In summary, *S. lugdunensis* is a common inhabitant of healthy human skin, especially in moist areas, with colonization rates suggesting it is an integral part of the skin microbiome.

The transition of *S. lugdunensis* from a commensal organism to a pathogenic one is influenced by several factors, including its inherent virulence mechanisms, environmental conditions, and host factors [1]. *S. lugdunensis* possesses unique virulence traits that differentiate it from other coagulase-negative staphylococci (CoNS) [1,34]. These include the ability to produce biofilms, which facilitate adherence to host tissues and medical devices, and the secretion of various virulence factors such as fibrinogen-binding proteins and autolysins. These factors contribute to its pathogenic potential, particularly in the context of invasive infections like endocarditis and skin infections. The switch can also be influenced by environmental stressors, such as changes in the skin microbiota or breaches in the skin barrier [51,52]. For instance, when the skin is compromised (e.g., due to injury, surgery, or the presence of medical devices), *S. lugdunensis* can exploit these opportunities to invade deeper tissues and cause infections. The immune status of the host plays a crucial role in determining whether *S. lugdunensis* remains a commensal or becomes pathogenic. Immunocompromised individuals or those with underlying health conditions are at a higher risk of developing infections from this organism [1,50,52]. The ability of *S. lugdunensis* to evade immune responses, through mechanisms such as the modulation of inflammatory responses, further enhances its potential to cause disease. The regulation of virulence gene expression is critical for the transition to a pathogenic state. The agr (accessory gene regulator) system, which is similar to that in S. aureus, plays a significant role in controlling the expression of virulence factors. The disruption of this regulatory system can lead to altered pathogenicity, indicating that genetic factors are essential in determining whether *S. lugdunensis* behaves as a commensal or a pathogen [34,50,51,52]. In summary, the switch from commensal to pathogenic states in *S. lugdunensis* is multifactorial, involving intrinsic virulence mechanisms, environmental conditions, host immune responses, and genetic regulation. Understanding these factors is crucial for developing strategies to manage infections caused by this opportunistic pathogen.

*S. lugdunensis* is increasingly recognized as an important pathogen in skin infections, rather than merely an opportunistic organism. While traditionally considered a part of the normal skin flora, recent studies have demonstrated its capability to cause significant skin and soft tissue infections (SSTIs), particularly in certain populations [1,23,40]. *S. lugdunensis* has been associated with various skin infections, including abscesses, cellulitis, and paronychia [40,52]. It is more virulent than other coagulase-negative staphylococci (CoNS) and has been implicated in serious infections that can occur in both healthy individuals and those with underlying conditions such as diabetes or immunocompromised states. A case series reported that *S. lugdunensis* was responsible for infections predominantly in the inguinal and perineal areas, with a high treatment success rate [40]. Another study highlighted that infections often present as cystic lesions or abscesses and that the organism can cause significant morbidity, especially in older adults [40]. While *S. lugdunensis* can act as an opportunistic pathogen, particularly in patients undergoing immunosuppressive therapies (like anti-TNF treatments), it can also initiate infections independently. For example, it has been shown to cause infections in patients without obvious predisposing factors [52]. In summary, *S. lugdunensis* is not merely an opportunistic pathogen but is important in the onset of skin diseases, particularly SSTIs. Its ability to cause infections in both healthy individuals and those with underlying health issues underscores its clinical significance. The emerging recognition of its pathogenic potential suggests that it should be considered a relevant pathogen in dermatological contexts, particularly for conditions such as hidradenitis suppurativa (HS) and other SSTIs.

In conclusion, the increased abundance of *S. lugdunensis* in HS can be attributed to the unique inflammatory and tissue-damaged environment of HS lesions, which supports bacterial colonization and persistence. *S. lugdunensis* employs various evasion mechanisms to circumvent immune responses, contributing to its pathogenicity. Further studies are needed to fully elucidate the immune responses against *S. lugdunensis* and to develop targeted therapies for managing HS associated with this bacterium.

Interestingly, in nearly half of the cases (49.6%), other bacteria were also isolated, indicating the potential for polymicrobial infections. Only a quarter of the cases (25.2%) were nosocomially acquired, suggesting that while *S. lugdunensis* can be a hospital-acquired pathogen, community-acquired infections are also significant. The study revealed a high cure rate of 100%, demonstrating the effectiveness of the current antimicrobial treatments. Most patients received systemic antibiotic therapy, with the majority treated with oral amoxicillin/clavulanic acid, cefuroxime axetil, or doxycycline. All isolates were susceptible to a range of antibiotics, including linezolid, moxifloxacin, rifampicin, teicoplanin, tigecycline, trimethoprim/sulfamethoxazole, and vancomycin. Resistance rates were low for most antibiotics, but notable resistance was observed for penicillin (28.5%) and clindamycin (36%). These resistance patterns align with previous data suggesting that while *S. lugdunensis* remains largely susceptible to many antibiotics, resistance to certain agents, particularly penicillin and clindamycin, is emerging [16,17].

The retrospective design of the study may introduce selection bias, potentially affecting the accuracy and reliability of the findings. Additionally, the single-center nature of the study limits the generalizability of the results to other settings and populations. Future research should adopt a multicenter approach to validate these findings across different healthcare environments and geographic regions. The sample size of 123 isolates, while sufficient for preliminary analysis, may not be large enough to detect less common resistance patterns or to perform more detailed subgroup analyses. Moreover, the study did not include long-term follow-up of patients, which is necessary to assess the durability of the treatment outcomes and the potential for recurrent infections. Information on patient comorbidities and other underlying health conditions was not comprehensively collected, which could influence infection outcomes and treatment efficacy. The study also did not account for prior antibiotic usage, which could influence resistance patterns observed in the bacterial isolates. A more detailed antibiotic history could provide insights into the development of resistance. Differences in treatment protocols and physician practices within the single center were not standardized, potentially introducing variability in patient outcomes that were not controlled for. The study relied on standard microbiological testing methods, which may not detect all resistance mechanisms or newer emerging resistance patterns. Advanced molecular techniques could provide a more comprehensive resistance profile. The focus on skin infections excluded other potential clinical manifestations of *S. lugdunensis*, such as endocarditis or osteomyelitis, which could provide a more complete understanding of the bacterium’s pathogenic potential. Additionally, the reliance on medical records and reported data may introduce reporting bias, where certain cases or outcomes are more likely to be documented than others. By addressing these limitations, future research can build on these findings and contribute to a more robust understanding of *S. lugdunensis* in skin infections.

## 5. Conclusions

In conclusion, this study characterized the clinical and microbiological profile and treatment outcomes of a large series of patients from whom *S. lugdunensis* was isolated from skin infections at a single center. Continued surveillance and antimicrobial stewardship are essential to monitor resistance trends and ensure effective management of *S. lugdunensis* infections.

## Figures and Tables

**Table 1:** Clinical characteristics of 123 patients with *Staphylococcus lugdunensis* isolated from skin samples.

**Gender**	
Male N, %	62/123 (50.4%)
Female N, %	61/123 (49.6%)
**Age**, years Mean	40.24
Median ± SD	38 ± 20.14
**Age groups**	
From 0- to 18-year-old	12/123 (9.8%)
Above the age of 66-year-old	15/123 (12.2%)
**Sample type**	
Exudate	16/123 (13%)
Pus	104/123 (84%)
Tissue	3/123 (2.4%)
**Sample site**	
Head and neck	14/123 (11.4%)
Upper limbs	18/123 (14.6%)
Trunk	22/123 (17.9%)
Buttocks	21/123 (17.1%)
Inguinal fold/genital area	15/123 (12.2%)
Lower limbs	33/123 (26.8%)
**Body site**	
Above waist	41/123 (33.7%)
Below waist	82/123 (66.7%)
**Trauma history, N, %**	7/123 (5.7%)
**Clinical presentation**	
Abscess	18/123 (14.6%)
Hidradenitis suppurativa (HS)	32/123 (26%)
Pilodinal cyst	4/123 (3.3%)
Folliculitis	17/123 (13.8%)
Cellulitis	10/123 (8.1%)
Wound/ulcer	13/123 (10.6%)
Intertrigo	6/123 (4.9%)
Psoriasis	6/123 (4.9%)
Eczema	4/123 (3.3%)
Acne	8/123 (6.5%)
Dissecting cellulitis	1/123 (0.8%)
Lymphoma	1/123 (0.8%)
Erythema multiforme	2/123 (1.6%)
**Other bacteria isolated**	
No other bacteria were isolated	62/123 (50.4%)
Yes, other bacteria were also isolated	61/123 (49.6%)
**Hospital acquired**	
No	92/123 (74.8%)
Yes	31/123 (25.2%)
Immunological status	
Immunosuppressed	38/123 (30.9%)
Immunocompetent	85/123 (69.1%)
**Treatment**	
Topical treatment	43/123 (35%)
Systemic antibiotic treatment	80/123 (65%)
**Type of treatment**	
Topical fusidic acid	21/123 (17.1%)
Topical mupirocin	10/123 (8.1%)
Topical clindamycin	12/123 (9.8%)
Oral cefuroxime axetil	19/123 (15.4%)
Oral amoxicillin/clavulanic acid	28/123 (22.8%)
Oral doxycycline	18/123 (14.6%)
Oral minocycline	15/123 (12.2%)
**Treatment outcome**	
	123/123 (100%)

**Table 2:** Other bacteria isolated from the 123 skin specimens positive for *Staphylococcus lugdunensis*.

*Staphylococcus aureus*
*Staphylococcus caprae*
*Staphylococcus cohnii*
*Staphylococcus epidermidis*
*Staphylococcus haemolyticus*
*Staphylococcus hominis*
*Staphylococcus intermedius*
*Mammaliicoccus sciuri* (*Staphylococcus sciuri*)
*Staphylococcus simulans*
*Staphylococcus warneri*
*Acinetobacter Iwofii*
*Achromobacter denitrificans*
*Corynebacterium amycolatum*
*Cutibacterium acnes* (*Propionibacterium acnes*)
*Enterobacter aerogenes*
*Enterobacter cloacae*
*Enterococcus faecalis*
*Escherichia coli*
*Finegoldia magna*
*Gemella haemolysans*
*Klebsiella oxytoca*
*Klebsiella pneumoniae*
*Peptoniphilus asaccharolyticus*
*Peptrostreptococcus prevotii*
*Prevotella intermedia*
*Proteus mirabilis*
*Pseudomonas aeruginosa*
*Pseudomonas stutzeri*

**Table 3:** Antibiotic susceptibilities of 123 *Staphylococcus lugdunensis* isolates from skin samples.

	Sensitive N (%)	Resistant N (%)
Penicillin	88/123 (71.5%)	35/123 (28.5%)
Clindamycin	87/123 (70.7%)	36/123 (36%)
Erythromycin	91/123 (74%)	32/123 (26%)
Fosfomycin	118/123 (95.9%)	5/123 (4.1%)
Gentamicin	118/123 (95.9%)	5/123 (4.1%)
Levofloxacin	121/123 (98.4%)	2/123 (1.6%)
Linezolid	123/123 (100%)	0/123 (0%)
Moxifloxacin	123/123 (100%)	0/123 (0%)
Nitrofurantoin	123/123 (100%)	0/123 (0%)
Oxacillin	117/123 (95.1%)	6/123 (4.9%)
Rifampicin	123/123 (100%)	0/123 (0%)
Teicoplanin	123/123 (100%)	0/123 (0%)
Tetracycline	115/123 (93.5%)	8/123 (6.5%)
Tigecycline	123/123 (100%)	0/123 (0%)
Tobramycin	114/123 (92.7%)	9/123 (7.3%)
Trimethoprim/Sulfamethoxazole	123/123 (100%)	0/123 (0%)
Vancomycin	123/123 (100%)	0/123 (0%)
Fusidic acid	108/123 (87.8%)	15/123 (12.2%)

N: number.

## Data Availability

The data presented in this study are available on request from the corresponding author.
